# Nonparametric assessment of mangrove ecosystem in the context of coastal resilience in Ghana

**DOI:** 10.1002/ece3.10388

**Published:** 2023-07-31

**Authors:** Daniel Aja, Michael Miyittah, Donatus Bapentire Angnuureng

**Affiliations:** ^1^ Africa Center of Excellence in Coastal Resilience, Center for Coastal Management University of Cape Coast Cape Coast Ghana; ^2^ Department of Environmental Science University of Cape Coast Cape Coast Ghana

**Keywords:** complex wetland, Ghana, Hazard index, InVEST, LULC, QADI

## Abstract

Cloud cover effects make it difficult to evaluate the mangrove ecosystem in tropical locations using solely optical satellite data. Therefore, it is essential to conduct a more precise evaluation using data from several sources and appropriate models in order to manage the mangrove ecosystem as effectively as feasible. In this study, the status of the mangrove ecosystem and its potential contribution to coastal resilience were evaluated using the Google Earth Engine (GEE) and the InVEST model. The GEE was used to map changes in mangrove and other land cover types for the years 2009 and 2019 by integrating both optical and radar data. The quantity allocation disagreement index (QADI) was used to assess the classification accuracy. Mangrove height and aboveground biomass density were estimated using GEE by extracting their values from radar image clipped with a digital elevation model and mangrove vector file. A universal allometric equation that relates canopy height to aboveground biomass was applied. The InVEST model was used to calculate a hazard index of every 250 m of the shoreline with and without mangrove ecosystem. Our result showed that about 16.9% and 21% of mangrove and other vegetation cover were lost between 2009 and 2019. However, water body and bare land/built‐up areas increased by 7% and 45%, respectively. The overall accuracy of 2009 and 2019 classifications was 99.6% (QADI = 0.00794) and 99.1% (QADI = 0.00529), respectively. Mangrove height and aboveground biomass generally decreased from 12.7 to 6.3 m and from 105 to 88 Mg/ha on average. The vulnerability index showed that 23%, 51% and 26% of the coastal segment in the presence of mangrove fall under very low/low, moderate and high risks, respectively. Whereas in the absence of mangrove, 8%, 38%, 39% and 15% fall under low, moderate, high and very high‐risk zones, respectively. This study will among other things help the stakeholders in coastal management and marine spatial planning to identify the need to focus on conservation practices.

## 1 INTRODUCTION

For natural capital accounting, structural factors such as height of mangrove canopy and biomass density are important considerations. They also provide soft defence for flood protection and play significant roles in controlling the global carbon balance to combat climate change (Houghton, 2005). Globally, deforestation of mangrove forest has been noted as a major source of greenhouse gas emissions (IPCC, 2007) and as a danger to the ecological services that mangroves supply (Aja et al., 2022). The evaluation of plans intending to lessen the effects of climate change using natural solutions requires accurate quantification and monitoring of temporal and spatial variations in mangrove cover and biomass (Omar et al., 2014).

Fatoyinbo and Simard (2012) estimated that mangroves cover about 7600 ha along the coast of Ghana and support coastal populations who are at risk of disasters by providing important ecosystem services such as natural coastline protection, coastal erosion reduction, improvement of water quality and provision of alternative livelihoods (Aja et al., 2022; Mondal et al., 2018).

The ecosystem services provided by mangroves in Ghana are essential to the well‐being of both local communities and the broader environment. Protecting and restoring these important ecosystems is therefore crucial for sustainable development in the region.

Mangrove assessment in the context of coastal resilience involves evaluating the role of mangrove ecosystems in promoting the ability of coastal areas to withstand and recover from natural disasters and other environmental stresses (Sebesvari et al., 2019; Whelchel et al., 2018). This assessment typically involves a range of factors, including the extent and diversity of mangrove ecosystems, the degree of protection they provide against storm surges and erosion and their ability to buffer against other climate‐related impacts (Estrella & Saalismaa, 2013; Sudmeier‐Rieux et al., 2021). To assess the role of mangroves in promoting coastal resilience, researchers have used a variety of methods, including remote sensing, field surveys and modelling.

It has been underlined how crucial remote sensing is in this context as one of the key sources of spatial data (Lagomasino et al., 2016). Given the complexity of the tropical mangrove forest ecosystem, remote sensing methods are not without their limits when assessing mangrove ecosystem (Son et al., 2015). The tropical environment's complex mangrove forest ecosystem, cloud cover and saturation at particular biomass levels make it difficult to use optical satellite technology only for mangrove monitoring and assessment (Aja et al., 2022). The closed canopy structure of tropical mangrove forests and the fact that spectral signature is insensitive to the spatial changes of aboveground biomass (AGB) greater than 150 Mg/ha may be the reason for the lack of effectiveness of optical remote sensing in mangrove structural assessment (Omar et al., 2014).

Aboveground biomass is estimated differently through a backscatter mechanism using active remote sensing techniques such as synthetic aperture radar (SAR). The ability of SAR technology to penetrate through closed tree canopy has contributed to the recent advances in mangrove assessment and monitoring (Omar et al., 2014). As SAR is one of the practical ways to acquire remote sensing data without the effect of cloud cover and independent of weather or light conditions, interest in radar remote sensing for monitoring tropical mangrove forests is developing (Thomas et al., 2017). LiDAR is another method with the ability to precisely capture the structure of mangroves without weather interference (Tianyu et al., 2020), but the lack of time series data with global coverage limits its application on a continental to global scale (Hu et al., 2016).

Many researchers are now incorporating radar data from various sensors for a more accurate quantification of mangrove extent and other biophysical properties due to the recent availability of active satellite data with increasing spatiotemporal coverage (De Santiago et al., 2013; Pinki et al., 2019; Thomas et al., 2018). The majority of satellite estimations of AGB were generated from the shuttle radar topographic mission digital elevation model (SRTM DEM) that was produced in the year 2000 (Fatoyinbo and Simard, 2013; Giri et al., 2011). The digital elevation model can be combined with synthetic aperture radar images, such as ALOS PALSAR data, to further improve the satellite estimations of aboveground biomass density (Omar et al., 2014).

Conventional mapping methods have been employed by many researchers to provide information on mangrove structure, but these methods are constrained by the availability of images, the requisite computational power and the required technical know‐how (Gorelick et al., 2017; Yancho et al., 2020). In particular for mangrove mapping and monitoring, new techniques and technology such as cloud computing are ushering in a new era (Wang et al., 2019; Wulder et al., 2018; Yancho et al., 2020). Platforms for geospatial data processing on the cloud, such as Google Earth Engine (GEE), give users unparalleled access to a vast library of ready‐to‐use geospatial data (Gorelick et al., 2017; Yancho et al., 2020).

Google Earth Engine eliminates the requirement for local standalone computers to download and process data by storing data locally and performing client‐versus‐server operations (Amani et al., 2020; Yancho et al., 2020). This resolves issues with hardware and the requirement for technical know‐how necessary for remote sensing. These advancements enable the rapid development and application of different mapping techniques across various spatial scales, opening up advanced remote sensing applications to more users (Gorelick et al., 2017; Hansen et al., 2013). Additionally, GEE open‐source nature allows for flexibility and customization while facilitating methodological repeatability (Vos et al., 2019). Currently, a number of studies have used GEE to map mangrove AGB and shown its efficiency, offering encouraging advancements over earlier techniques. Carreiras et al. (2013) used L‐band synthetic aperture radar coupled with field data to estimate AGB and reduced uncertainties. Fatoyinbo and Simard (2013) used a combination of mangrove map derived from Landsat, lidar canopy height estimates and elevation data from SRTM to derive the structure and biomass of mangroves for the African continent.

Assessing coastal vulnerabilities and the potential for mangrove ecosystem to contribute towards the protection of coastal communities is essential for effective long‐term planning, sustainability and the management of coastal resilience (Al Ruheili & Boluwade, 2023). The Integrated Valuation of Ecosystem Service and Trade‐offs (InVEST) Coastal Vulnerability model has been applied elsewhere to assess coastal vulnerability and to explore the role of natural habitats such as mangroves to mitigate coastal hazards (Arkema et al., 2013; Aneseyee et al., 2022; Ai et al., 2022; Al Ruheili & Boluwade, 2023). Mangrove ecosystems must be accurately mapped and inventoried in order to determine how they are changing and to what pressures they are subjected to, before their potential role as a natural defence can be assessed. Such information serves as the foundation for in‐depth social‐ecological assessments that can guide efforts to lessen anthropogenic pressures on mangrove and encourage sustainability and conservation.

Few researchers have combined optical and radar data for improved mapping and assessment of mangroves (Ghorbanian et al., 2021; Yancho et al., 2020); however, such research as well as information on the role of natural habitats in protecting coastal areas is limited in Ghana. In this study, we used GEE to combine optical and radar data set in a spatial framework to monitor and evaluate mangrove ecosystem. We employed InVEST model to simulate the degree to which mangrove ecosystem contributes to coastal resilience. The aim of this article was to analyse and evaluate mangrove ecosystem with a view to determine the impact of human‐induced pressure on the temporal and spatial variation of mangrove extent and AGB density as well as to demonstrate the importance of the mangrove ecosystem as a robust nature‐based solution to coastal disasters. The research questions that this study intends to address are: (1) What is the impact of human‐induced pressure on the temporal and spatial variation of mangrove extent and aboveground biomass (AGB) density? and (2) How does this affect the ability of mangrove ecosystems to serve as nature‐based solutions to coastal disasters?

## 2 MATERIALS AND METHODS

### 2.1 Description of study site

**FIGURE 1:**
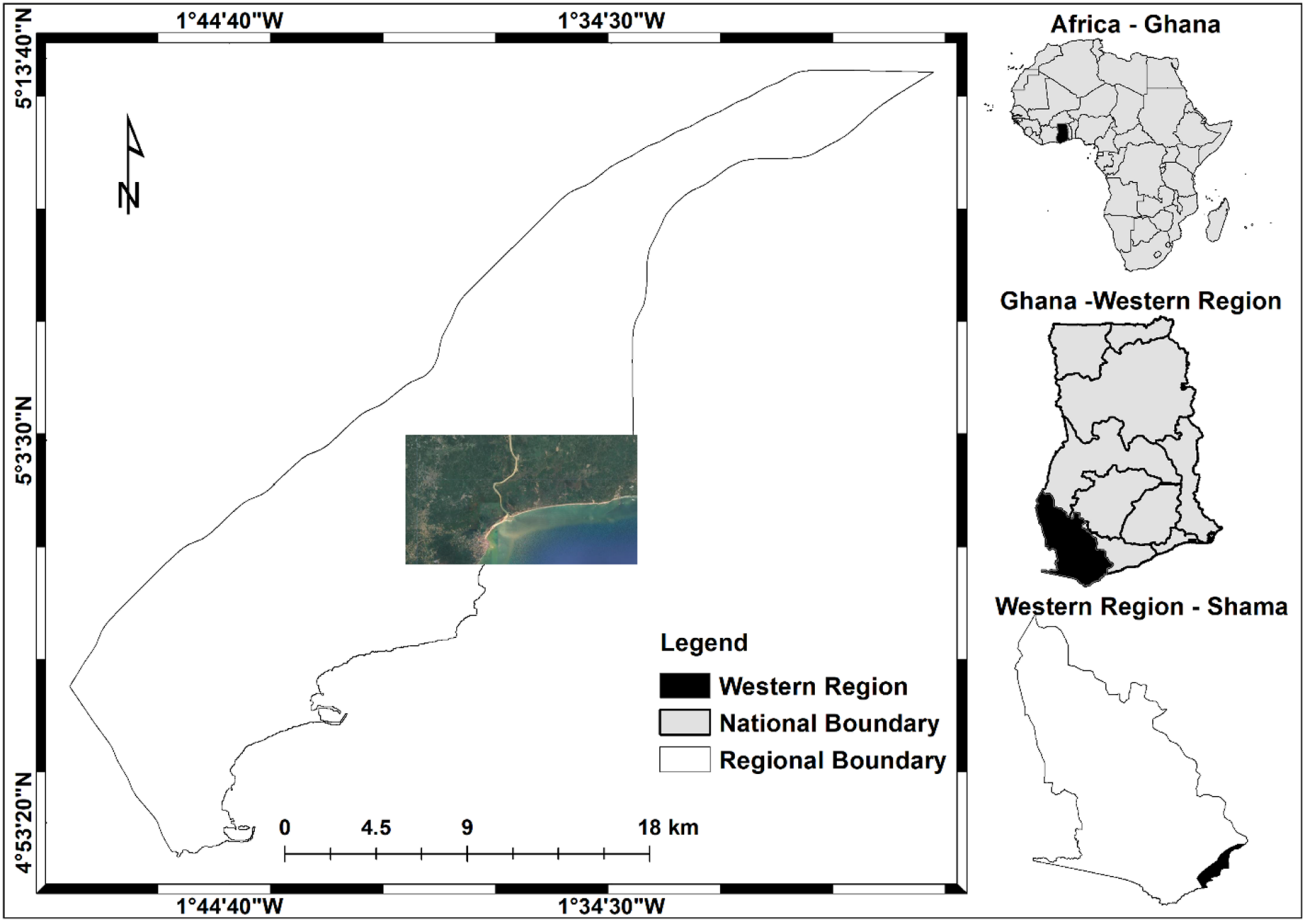
Study location map.

This study was conducted at a complex wetland fringing part of Anlo community, Shama district, Western region, Ghana (Figure 1). The 52 km^2^ delineated area is bounded within latitude 5°1′30″ N and 5°3′5″ N, longitudes 1°34′30″ W and 1°37′30″ W. The wetland is situated at the lower part of the Pra watershed with an area of 23,000 km^2^ (Opoku‐Ankomah, 2000) and occupies roughly 20% of the landmass of Ghana (Owusu et al., 2015). In terms of hydrology, this area is within the floodplains of Pra River, which has a direct link with the ocean.

The study area witnesses yearly high temperature ranging from 21.74°C to 31.6°C (Bessah et al., 2018) with a monthly average relative humidity above 70% (Kwabena et al., 2013) and a long‐term average rainfall of 1446 mm (Bessah et al., 2019), making the climatic condition a tropical monsoon (Am) (Kottek et al., 2006). The dominant soil types in the upper part of the basin are Acrisols and Lixisols, which are characterized by fairly high possibility for run‐off (Ross et al., 2018), exposing the low parts of the watershed to water disasters during periods of heavy rainfall (Bessah et al., 2020).

The elevation is very low (0–42 m), the coastline is irregular with a sandy beach next to the wetland and the ocean is marked by pounding surf (Friends of the Nation, 2014). In the past 50 years, the shoreline has eroded an average of 100 m (Coastal Resources Center/Friends of the Nation., 2010). The most common mangrove genera in the wetland are *Rhizophora*, *Avicennia* and *Laguncularia* and saltwater grass *Paspalum vaginatum* (*Poaceae*) is the major plant in the adjacent wetlands (Okyere, 2018). The community demography is made up of 1028 males and 1203 females who are predominantly fisherfolks (Coastal Resources Center/Friends of the Nation., 2010). The community heavily depends on the mangrove ecosystem for a variety of activities, which intensifies the dynamics of land use change (Aja et al., 2022).

### 2.2 Data requirement and sources

ALOS PALSAR‐2 data for 2009 were retrieved from the JAXA website at http://www.eorc.jaxa.jp/ALOS/en/palsarfnf/fnfindex.htm. These data are a dual‐polarized L‐band SAR with a 25‐m resolution, 10^0^ × 10^0^ in longitude and latitude. The image collection ID is N06W002_09_sl_HH, N06W002_09_sl_HV. The Google Earth Engine database, which is accessible at https://developers.google.com/earth‐engine/guides/sentinel1, was used to retrieve the Sentinel‐1 data for 2019. These data are a C‐Band synthetic aperture radar with a descending pass, dual‐polarized with a 25 ‐m resolution in interferometric wide swath mode. ee.ImageCollection (‘COPERNICUS/S1_GRD’) is the Image Collection ID. The database of the GEE was used to retrieve the 2009 Landsat 7 surface reflectance tier1 data. Four visible and near‐infrared bands, two short wave infrared bands and one thermal infrared band are all present in this data. ee.ImageCollection (‘LANDSAT/LE07/C01/T1_SR’) is the image collection ID: More information about this data can be found at https://www.usgs.gov/landsat‐missions/landsat‐surface‐reflectance.

Landsat 8 surface reflectance tier1 data (2019) were retrieve from GEE database. These data contain five visible and near‐infrared bands, two short wave infrared bands and two thermal infrared bands. The image ID is ee.ImageCollection (‘LANDSAT/LC08/C01/T1_SR’). Additional information about these data can be found at https://www.usgs.gov/landsat‐missions/landsat‐surface‐reflectance. The SRTM DEM data were retrieved from GEE database. These data are provided by NASA JPL at a resolution of ~30 m. ee.Image (‘USGS/SRTMGL1_003’) is the image ID. More information at NASA/USGS/JPL‐Caltech. The global mangrove distribution vector that was generated by the global mangrove watch (GMW) in 2010 was needed to delineate mangroves and was accessed at https://data.unep‐wcmc.org/datasets/45 (Giri et al., 2011). Global mangrove watch is a shapefile of global mangrove areas as at 2010. We used bathymetry from the general bathymetric chart of the global ocean (GEBCO) available at https://www.gebco.net/data, wavewatch III and continental shelf contour to assess coastal vulnerability. The GEBCO bathymetry has a spatial resolution of 15 arc sec. Information about wavewatch III and continental shelf contour can be found at Coastal Vulnerability Model—InVEST documentation (storage.googleapis.com).

### 2.3 Methods

For this work, mangrove evaluation and monitoring were made using Google Earth Engine (GEE), a cloud‐based technology. To study the spatial changes in mangrove cover over time, LULC maps were made by jointly classifying SAR and optical data for two periods, namely 2009 and 2019. Mangrove aboveground biomass (AGB) as at year 2000 was estimated using basal area‐weighted height estimates from the SRTM DEM (Simard et al., 2019). The AGB estimates from satellite was compared with field measurements to examine the temporal and spatial variation over time.

#### 2.3.1 Mangrove extent mapping

**FIGURE 2:**
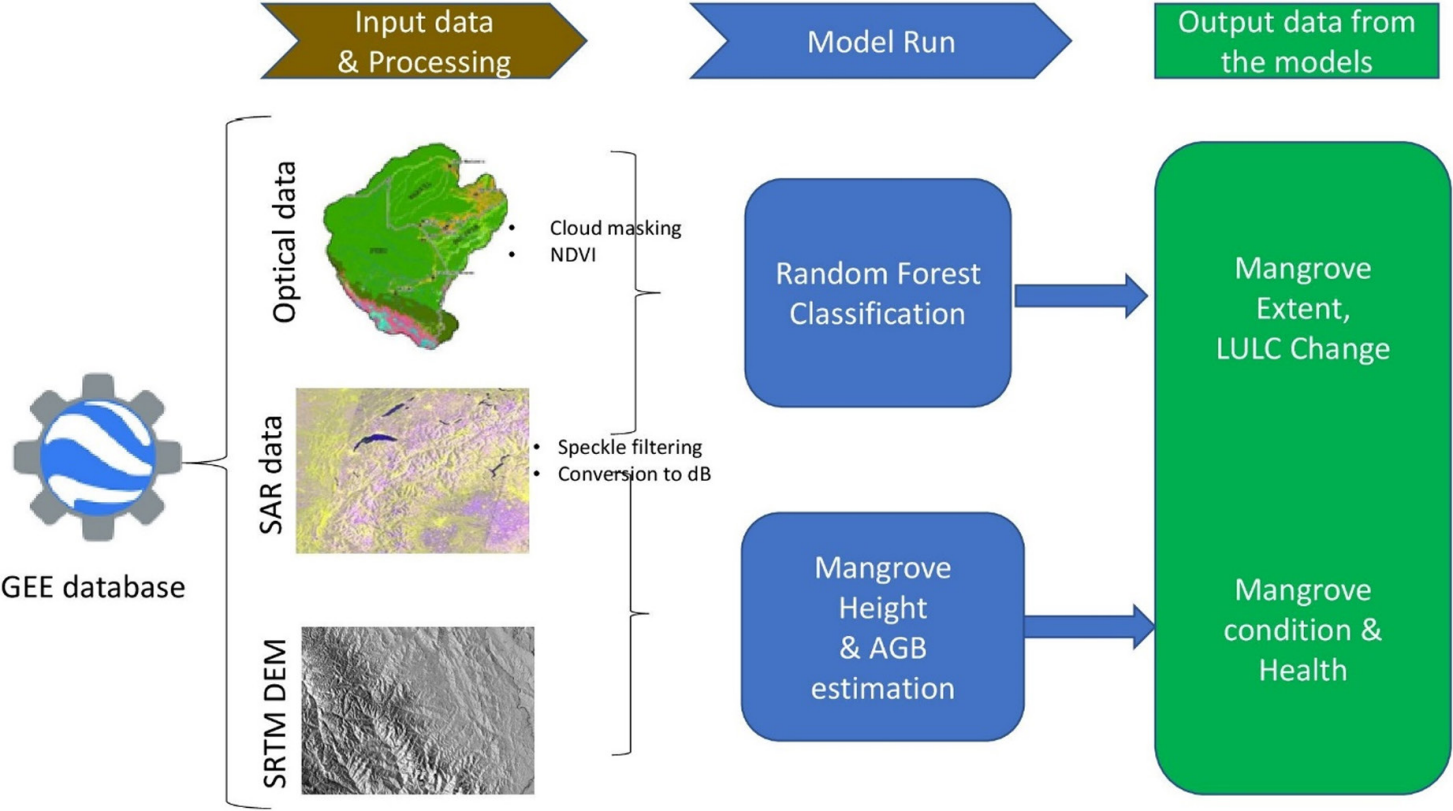
Flow chart of the methodological approach.

The method involves retrieving the following data set: Sentinel‐1 data, ALOS PALSAR‐1 and 2 data, Landsat 8 and Landsat 7 surface reflectance tier1 data, and the mangrove distribution vector from GEE database as shown in Figure 2. These data were loaded into the Google Earth Engine code editor and then these data were preprocessed using cloud masking for the optical images (Giri et al., 2011; Hansen et al., 2013) and SAR speckle filtering to reduce speckle noise (Ayman et al., 2017). The images were enhanced by creating a function that masks out cloud shadows from optical images and another function was created that filters the speckle noise from SAR images.

The ALOS PALSAR‐1 and 2 data were converted from log_10_ to dB (decibels) using the formula below (JAXA, 2006).

(1) $$\gamma^{O}=10\mathrm{lo}g_{10}\left\langle {\mathrm{DN}}^{2}\right\rangle +\mathrm{CF}$$

where $\gamma^{O}$, Sigma naught (dB); DN, digital number; Calibration Factor (CF), 83.0 for the PALSAR data.

The NDVI (normalized difference vegetation index), which served as an information layer for the classifier, was used to transform the optical data into a composite image (Shi et al., 2016; Thomas et al., 2018).

#### 2.3.2 Random Forest (RF) algorithm and land use land cover change analysis

An ensembled tree‐based machine learning algorithm called random forest (RF) that employs decision trees to perform image classification was used. In this supervised classification, the algorithm builds a decision tree for each sample according to the predictors, the trees cast votes for each pixel to identify the type of land cover and the most supported value is given to each pixel. In order to ‘train’ the RF classifier, training samples must be created (Pelletier et al., 2016; Shelestov et al., 2017).

The methodology employed in this work is consistent with that suggested by Ghorbanian et al. (2021) and Yancho et al. (2020), and it entails gathering backscatter samples that represent each relevant landcover class and using the samples to train the classifier. With 100 trees and five predictors chosen at random for each split, the RF algorithm was run (Ghorbanian et al., 2021). Field campaigns that took place between December 2020 and April 2021 served as the basis for the training and validation data used in this work. To help with the creation of ‘training’ samples and result validation, high‐resolution satellite image of 2015 from google earth and a 2010 global mangrove distribution vector file were employed as data for reference.

Using GEE's built‐in ‘drawing tools’ feature, the region of interest (roi) was created and clipped to the data set. A polygon symbol was chosen from the drawing tools to add training samples to the speckle‐filtered VH image (Sentinel‐1). Water body, mangroves, bare/built‐up and other vegetation were identified as four distinct land cover classes. Each class was saved as a FeatureCollection with the name Landcover. The classes were merged to get one collection referred to as ‘new FeatureCollection’. The values of the backscatter for each selected landcover from the Sentinel‐1 image are included in the ‘new FeatureCollection’. Using this, the random forest classifier was ‘trained’. The ‘training’ data were overlaid on the defined optical and SAR images to be classified. The aforementioned procedure was made for 2009 and 2019 using SAR and optical images. The output was transferred to google drive to be processed further.

#### 2.3.3 Classification accuracy and result validation

The overall accuracy and the quantity allocation disagreement index (QADI) were calculated using the following formula:

(2) $$\text{Overall accuracy}=\frac{\text{Number of correctly classified pixels}\;\left(\mathrm{sum}\;\text{of diagonal}\right)}{\text{Number of total sampled pixel}}\times 100$$

(3) $$\text{QADI}=\sqrt{{\left(\frac{A}{N}\right)}^{2}+{\left(\frac{Q}{N}\right)}^{2}}$$

where, *N*, total number of sampled pixels; *A*, allocation disagreement of classes; *Q*, quantity disagreement of classes.

The quality allocation disagreement index (QADI) classification accuracy scale is as shown in Table 1. The quality allocation disagreement index is an efficient classification accuracy assessment for a variety of machine learning algorithms including random forest (RF). It has advantage over the traditional kappa coefficient because it solves some of the problematic paradoxes associated with Kappa (Feizizadeh et al., 2022).

**TABLE 1:** Quality allocation disagreement index (QADI) classification accuracy scale.

S/N	QADI scale	Strength of classification accuracy
1	0.00 ≤ QADI < 0.07	Very low disagreement (Very high confidence)
2	0.07 ≤ QADI < 0.12	Low disagreement (High confidence)
3	0.12 ≤ QADI < 0.20	Moderate disagreement (Moderate confidence)
4	0.20 ≤ QADI < 0.30	High level of disagreement (Low confidence)
5	0.30 ≤ QADI ≤ 1	lack of accuracy (Very low confidence)

Overall, 2131 training sample points were produced. The sample points were separated at random into ‘training’ and ‘validation’ groups of which 80 per cent of the points were used to ‘train’ the model, while 20 per cent were used for validation (Mahdianpari et al., 2020). As a result of using the same pixels for classifier training and validation, any systematic mistake was eliminated by this process (Geiß et al., 2017; Pimple et al., 2018; Shi et al., 2016). Utilizing 20% of the training sample, stratified random samples were created for an independent accuracy evaluation, which follows the procedure outlined by Barenblitt and Fatoyinbo (2020). High‐resolution satellite imagery that is accessible in ArcGIS was used to verify each point.

#### 2.3.4 Estimation of mangrove stand height

Mangrove canopy height is a key element in calculating aboveground biomass and carbon sequestration rates since it has a strong correlation with carbon turnover through litterfall production (Rovai et al., 2018; Saenger & Snedaker, 1993). In this study, mangrove canopy height estimate was made using SRTM digital elevation measurement (Farr et al., 2007) generated in February 2000. Mangrove height estimation was made following an approach that had had been effectively applied at regional scales (Fatoyinbo and Simard, 2013; Simard et al., 2006, 2008). The SRTM DEM values represent a height that lies between the elevation of the ground and the canopy's highest point (aka Lorey's height) (Lagomasino et al., 2016; Simard et al., 2006, 2008). The maximum height is estimated to be 1.697 × (times) this value based on empirical data from field measurements (Simard et al., 2019). We used the global mangrove distribution vector to isolate mangrove areas and mask nonmangrove regions in the SRTM elevation data set (Giri et al., 2011).

To estimate mangrove height, the SAR image (ALOS PALSAR‐1) and the SRTM elevation model were clipped to the global mangrove distribution vector file (Figure 2). This extracts the backscatter values for the areas where the baseline mangrove vector indicates that there are mangroves. These values and the SRTM elevation were used to estimate the maximum canopy height using a regression model that relates SRTM elevation measurements to maximum canopy height as described by Simard et al. (2019).

(4) $$\text{SRTM}\;H_{\max}=H_{\text{SRTM}}\times 1.697$$

where *H*_SRTM_, original SRTM DEM; SRTM *H*_max_, new maximum canopy height data set.

#### 2.3.5 Mangrove aboveground biomass estimation

Aboveground biomass (AGB) estimates can be produced using allometric equations once the height of the mangrove canopy has been determined (Comley & McGuinness, 2005). The present study estimates AGB based on the correlation between canopy height and mangrove biomass (Lucas et al., 2014; Simard et al., 2019). We adopted a universal AGB model, which was developed and validated by Simard et al. (2019) based on 331 field plots from across three continents including Africa.

(5) $$\text{Aboveground biomass}\;\left(B\right)=3.25\times {H_{\mathrm{ba}}}^{1.53}$$

where *H*_ba_, basal area‐weighted height (~1.08*SRTM).

#### 2.3.6 Field inventory

Mangrove biomass is measured either directly through destructive harvesting of specific trees or indirectly through measurements of tree diameters and inference using allometric relationships (Chave et al., 2005). The destructive approach requires cutting down the trees, but the nondestructive approach uses allometric equations (Gibbs et al., 2007). Allometric equations, however, frequently depend on the locality and tree species (Chave et al., 2014; Smith & Whelan, 2006; Vashum & Jayakumar, 2012).

For this study, ground‐based mangrove inventory data were gathered using systematic random sampling and indirect estimation method. Field work was carried out between December 2020 and April 2021. A 100‐m transect was laid perpendicular to the shoreline using measuring tape. Four distinct 5 m × 5 m plots were set up 25 m apart along the transect for sampling. All sampling points' coordinates were recorded using Garmin 64 s GPS. For all mangrove species in all plots above 2 m, measurements of height and diameter at breast height (DBH) were taken. Using a calibrated long pole and a digital caliper, the heights and the DBH of the trees of each species were measured.

Allometric equation is used in the current study to determine the amounts of aboveground biomass for individual trees in the sample plots. Total biomass was calculated by adding up all of the tree biomass values in each plot along the transect. Biomass per plot was then expressed milligramme per hectare (Mg/ha). Site‐specific allometric models are uncommon in mangrove forests; hence, universal models are more frequently employed. For this study, AGB was estimated using two different universal allometric equations (Chave et al., 2005; Komiyama et al., 2005) and a regional allometric equation (Njana et al., 2016), which were developed specifically for mangrove, because there is no published site‐specific allometry for the study area. These allometries were chosen because wood density and height are their main driving factors (Fatoyinbo et al., 2018). The universal pantropic equation of Komiyama et al. (2005) is given below:

(6) $$\mathrm{AGB}=0.251\;{\text{34𝜌 34𝜌𝐷}}^{2.46}$$

where AGB, aboveground biomass (Mg per tree); 𝜌, wood density (g cm^−3^); D, diameter at breast height (cm). The universal equation of Chave et al. (2005) is given by equation:

(7) $$\mathrm{AGB}=0.0509\;{\text{34𝜌 34𝜌𝐷}}^{2}\;\text{34𝐻}$$

where AGB, aboveground biomass (Mg/tree), *𝜌*, wood density (g cm^−3^), *D*, diameter at breast height (cm); *H*, height (m).

The universal equation of Njana et al. (2016) is given by:

(8) $$\mathrm{AGB}=0.353\;{\text{34𝜌}}^{1.13}\;{\text{34𝐷}}^{2.08}\;{\text{34𝐻}}^{0.29}$$

where AGB, aboveground biomass (Mg per tree); *𝜌*, wood density (g cm^−3^); *D*, diameter at breast height (cm); *H*, height (m).

The data on wood density were compiled by Muhd‐Ekhzarizal et al. (2018) and Fatoyinbo et al. (2018) from the world agroforestry wood density database. The range of wood density values includes globally reported values. We used the average of the middle values (0.87) of the wood density presented in Table 2 because none of the indicated wood densities are particular to Ghana.

**TABLE 2:** Values for selected mangrove wood density.

Species	Wood density (g cm^−3^)		
	Low	Middle	High
*Avicennia marina*	0.79	0.81	0.85
*Bruguiera gymnorrhiza*	0.63	0.84	1.05
*Ceriops tagal*	0.87	0.97	1.09
*Heriteria littoralis*	0.83	0.98	1.23
*Lumnitzera racemosa*	0.75	0.88	0.97
*Rhizophora mucronata*	0.94	1.02	1.12
*Sonneratia alba*	0.62	0.78	1.00
*Xylocarpus granatum*	0.59	0.70	0.83

### 2.4 Coastal vulnerability assessment using InVEST model

**FIGURE 3:**
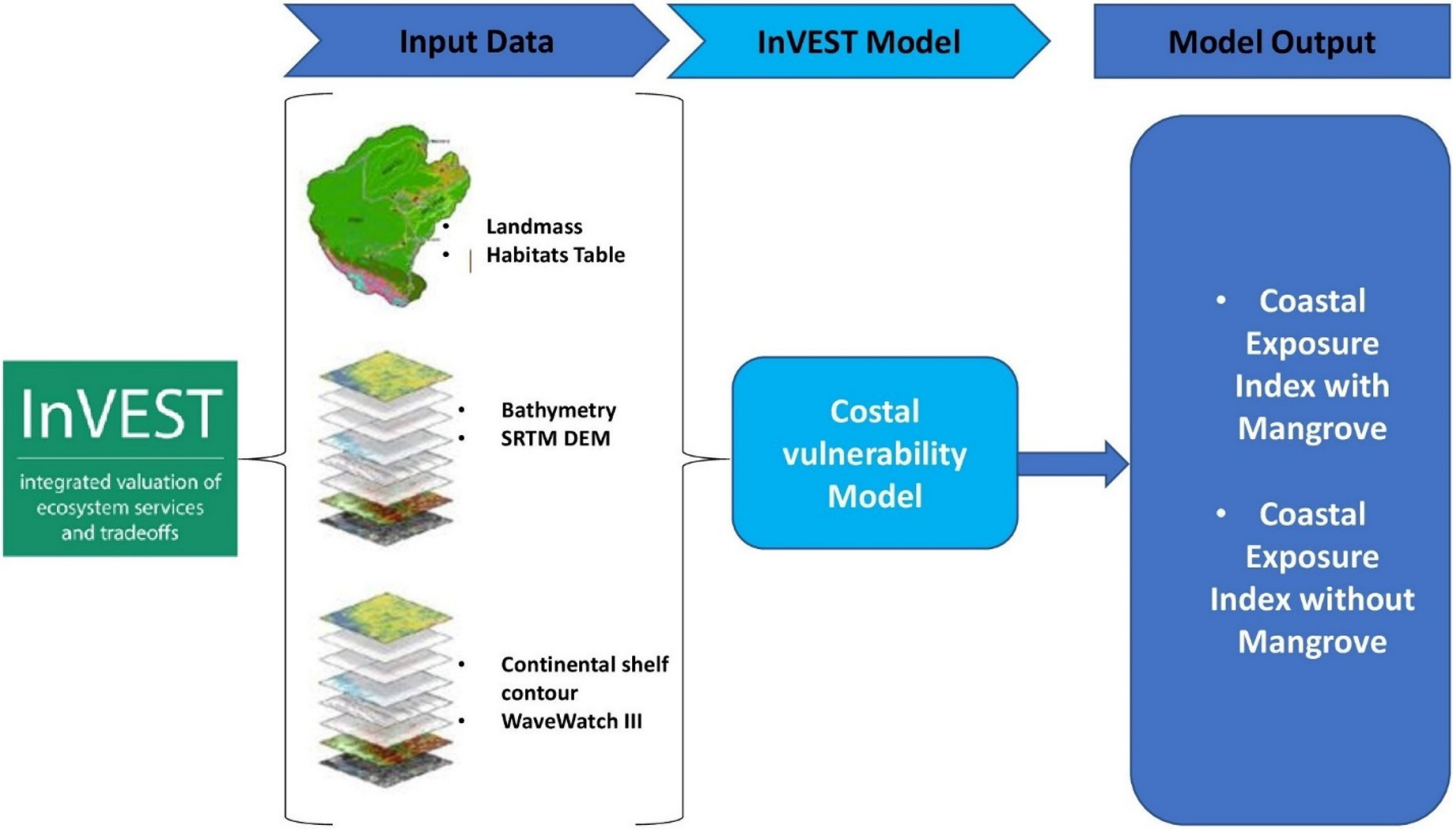
Coastal vulnerability modelling approach.

In order to compare the relative exposure of the coastlines with and without mangrove ecosystems, we estimated the coastal exposure index using the coastal vulnerability model in InVEST, available at www.naturalcapitalproject.org (Figure 3). The model is based on the findings of Hammar‐Klose and Thieler (2001), who investigated how ecosystems function to safeguard coastlines. The coastal vulnerability model considers climatic and biophysical variables such as wind, wave, surge, habitat, geomorphology and relief that influence coastal inundation and erosion.

The model computes the coastal exposure index (EI) for each coastline segment as the geometric mean of all the variable:

(9) $$\mathrm{EI}={\left(R_{\text{Geomorphology}}R_{\text{Relief}}R_{\text{Habitats}}R_{\text{Wind}}R_{\text{Wave}}R_{\text{Surge}}\right)}^{1/6}$$

where, *R* denotes the variable.

The approach combines the ranks of up to seven biological and physical variables at each point along the shoreline to calculate the coastal exposure index. The coastal exposure index is given a rank from 1 to 5 based on a combination of user‐ and model‐defined criteria. The ranks are: very low exposure (rank = 1), low exposure (rank = 2), moderate exposure (rank = 3), high exposure (rank = 4) and very high exposure (rank = 5).

## 3 RESULTS

### 3.1 Time series analysis of Forest change

**FIGURE 4:**
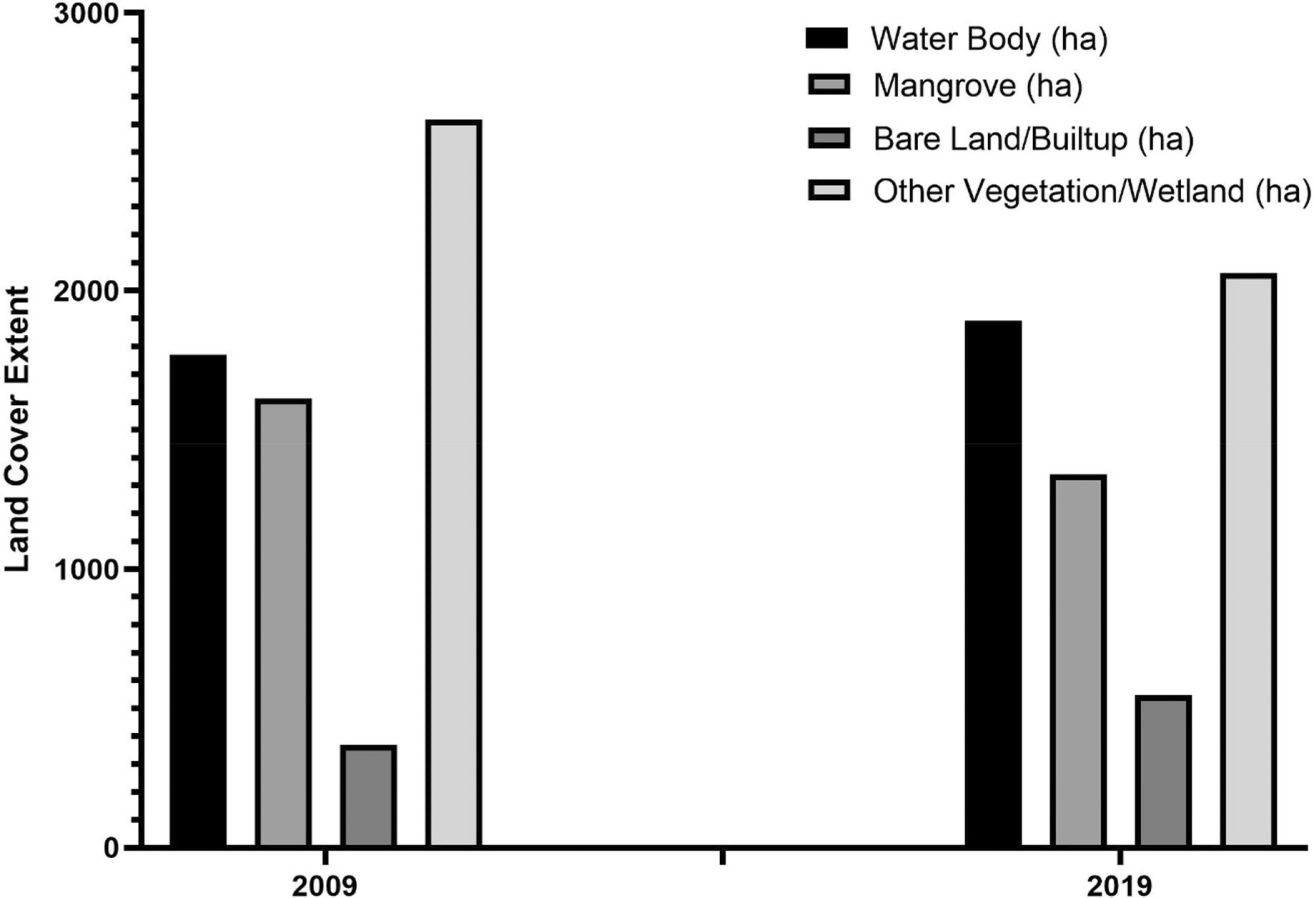
Changes in land use and cover between 2009 and 2019.

**FIGURE 5:**
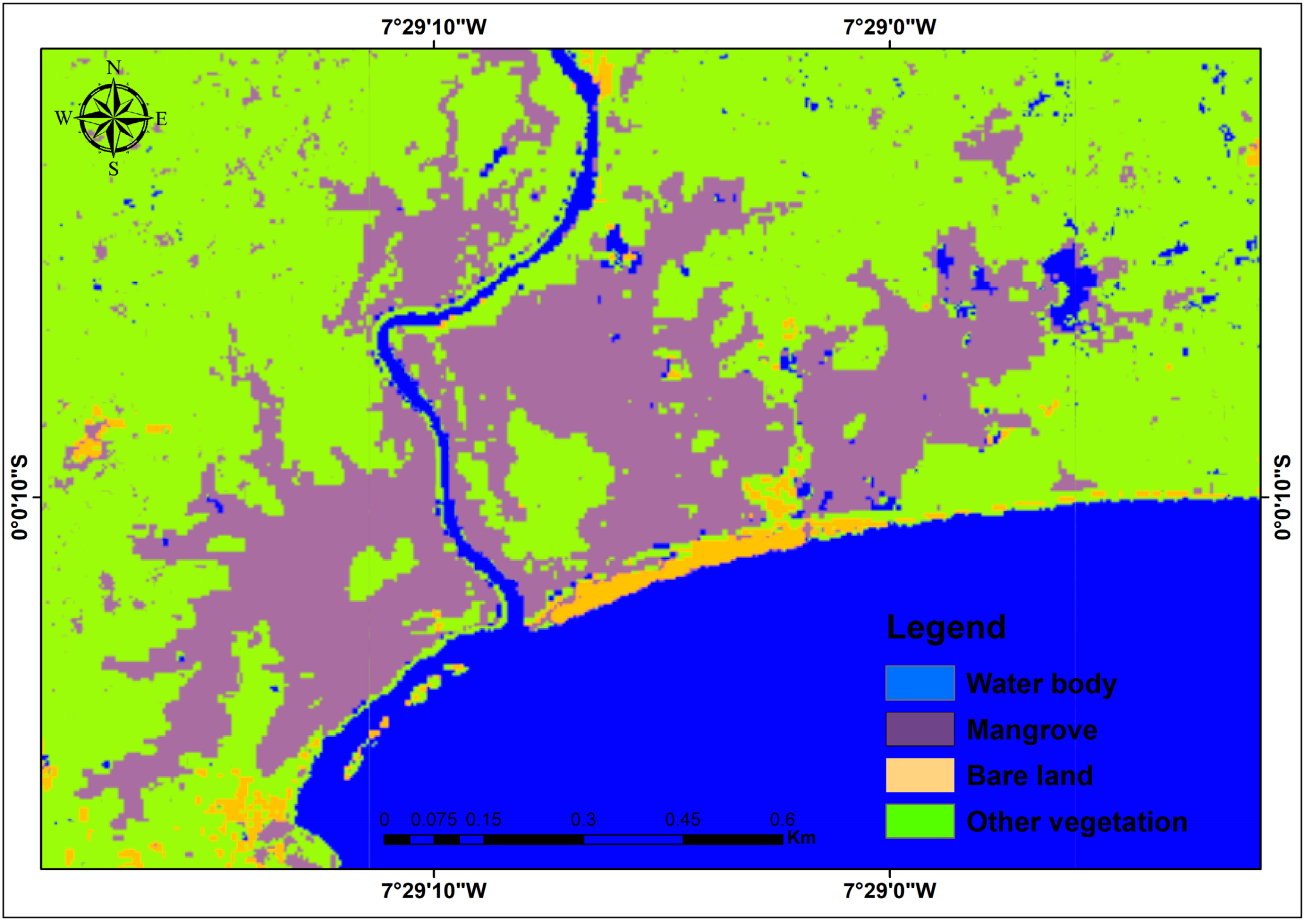
Map of land cover extent for 2009.

**FIGURE 6:**
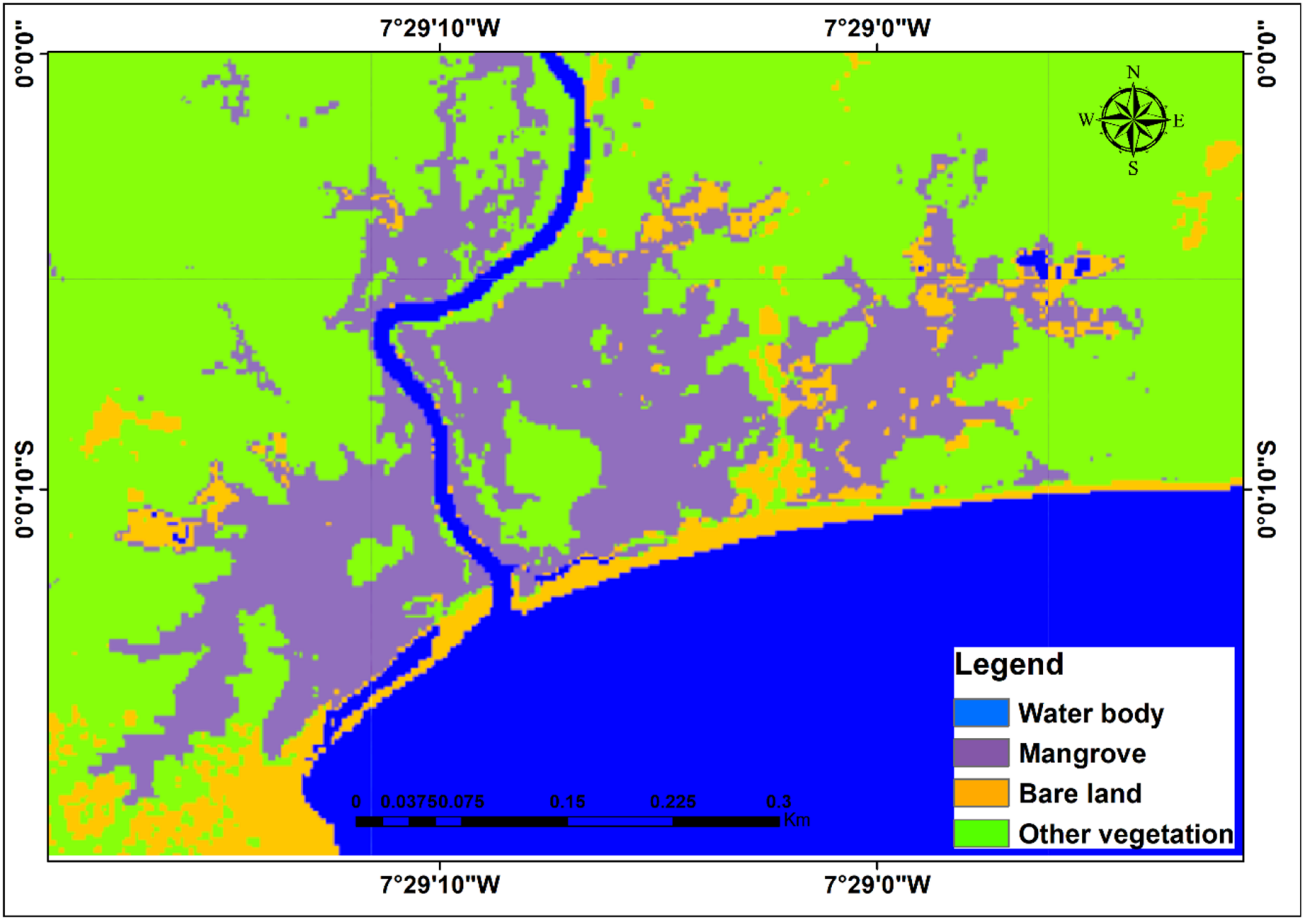
Map of land cover extent for 2019.

Several iterations were run utilizing optical and SAR data together until the model result were reasonably close to ground truth. This process was conducted for 2009 and 2019. Figures 4, 5 and 6 show the results of LULC changes for the two time periods. Our findings showed that mangrove area was 1340 ha, water body extent was ‘1891 ha’, bare/built‐up area was 549 ha and other vegetation was 2062 ha for Sentinel‐1 and Landsat 8 combined (2019 data). In contrast, the combined 2009 data from ALOS PALSAR‐2 and Landsat 7 showed that mangroves covered 1613 ha, water bodies covered ‘1770 ha’, bare/built‐up area covered 370 ha and other vegetation covered 2617 ha.

With a QADI of 0.00529, Sentinel‐1 and Landsat 8 both had an overall classification accuracy of 99.1%, whereas the combination of ALOS PALSAR‐2 and Landsat 7 had an overall classification accuracy of 99.6% and a QADI of 0.00794, respectively. The mangrove classification error matrix for each time period is displayed in Tables 3 and 4 while the range of quality allocation disagreement index (QADI) is shown in Table 1. The results revealed that using optical images in conjunction with synthetic aperture radar boosted the classification accuracy upto 99 per cent and decreased error of omission or commission of mangroves. The values of QADI for the two time period indicated very high agreement and confidence.

**TABLE 3:** Landcover classification confusion/error matrix using ALOS PALSAR‐2 and Landsat‐7 Imagery.

Classes	Water body	Mangroves	Bare/built‐up	Other vegetation	Row total	User's accuracy (%)
Water body	81	0	0	0	81	100
Mangroves	0	647	0	0	647	100
Bare/built‐up	0	1	26	5	32	81.3
Other vegetation	0	1	0	944	945	99.9
Column total	81	649	26	964	1705	
Producer's accuracy (%)	100	99.7	100	97.9		

*Note*: 99.6% overall accuracy and a QADI of 0.00794.

**TABLE 4:** Landcover classification confusion/error matrix using Landsat 8 and Sentinel‐1 imagery.

Classes	Water body	Mangrove	Bare/built‐up	Other vegetation	Row total	User's accuracy (%)
Water body	81	0	0	0	81	100
Mangrove	0	642	1	4	647	99.2
Bare/built‐up	0	0	32	0	32	100
Other vegetation	0	9	0	936	945	99
Column total	81	651	33	940	1705	
Producer's accuracy (%)	100	98.6	96.9	99.6		

*Note*: 99.1% overall accuracy and a QADI of 0.00529.

### 3.2 Accuracy assessment

**FIGURE 7:**
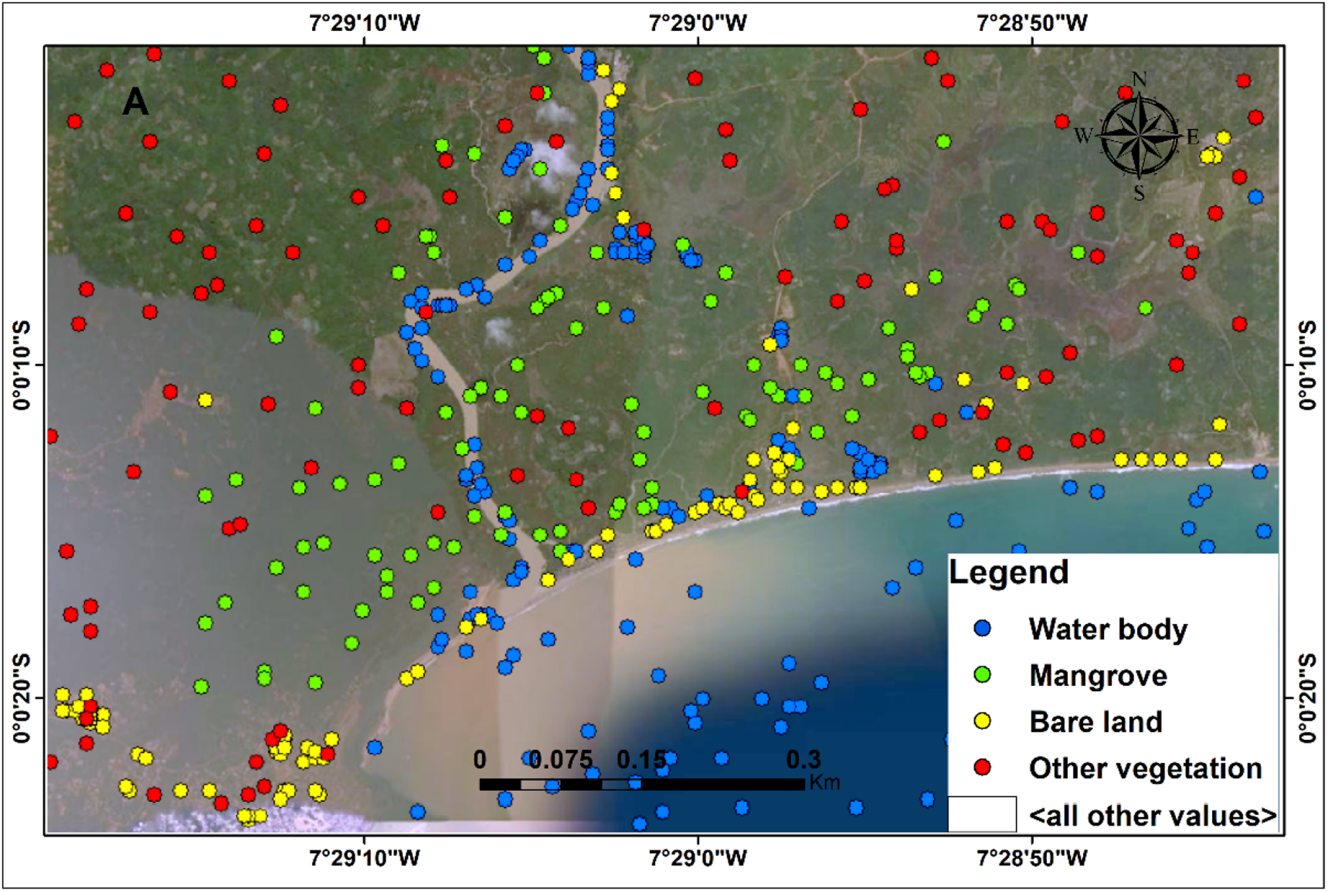
Independent accuracy assessment for 2009 land cover classification.

**FIGURE 8:**
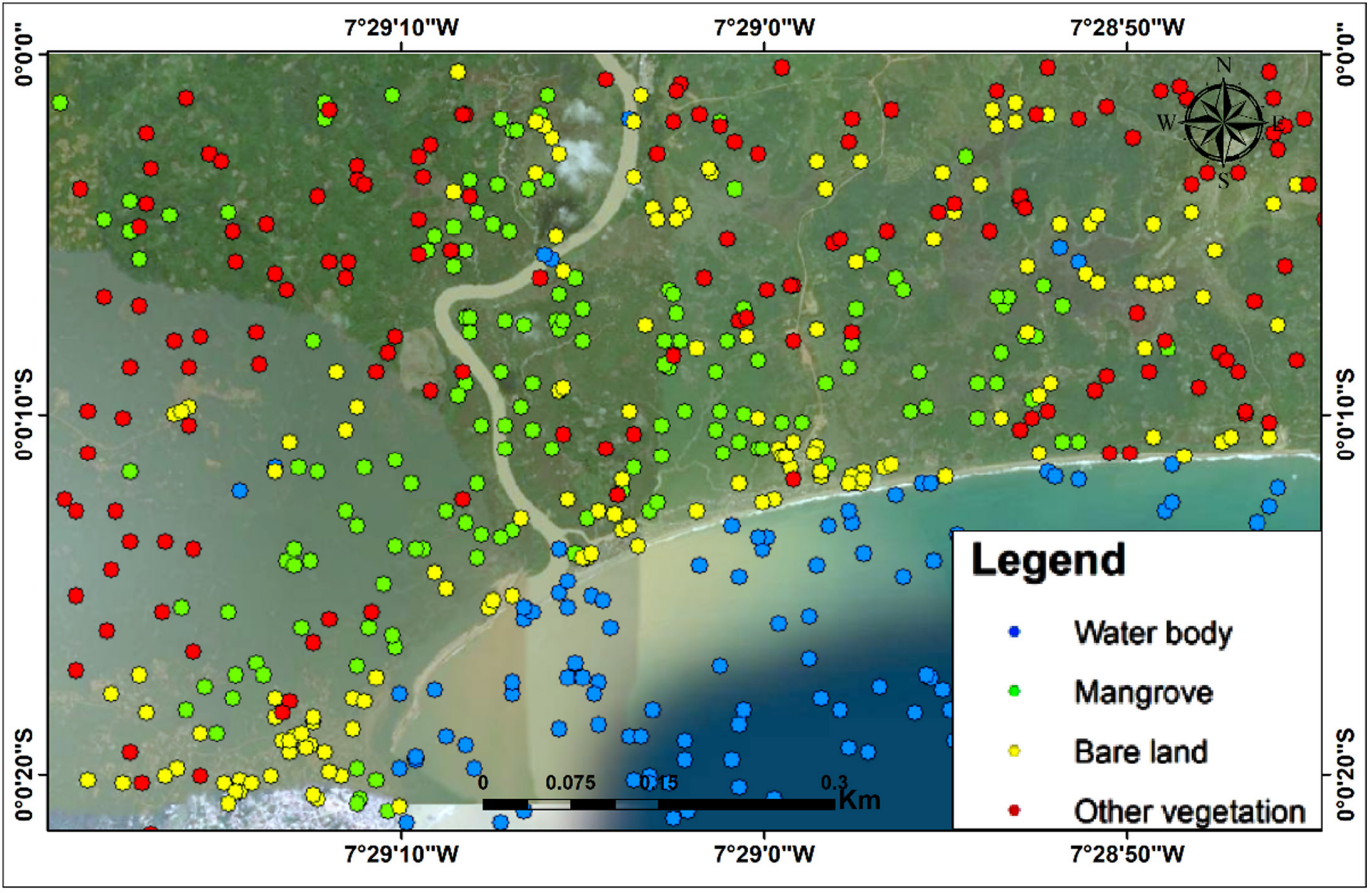
Independent accuracy assessment for 2019 land cover classification.

To offer thorough statistical analysis for each classification, confusion matrix and an independent accuracy assessment were used. The user and producer accuracy were quite high according to the confusion matrix for the two time periods (Tables 3 and 4). However, according to the independent accuracy assessment, somewhat higher accuracy was discovered for 2009, which made use of ALOS PALSAR‐2 and Landsat‐7 (Figures 7 and 8).

### 3.3 Estimation of canopy height

**FIGURE 9:**
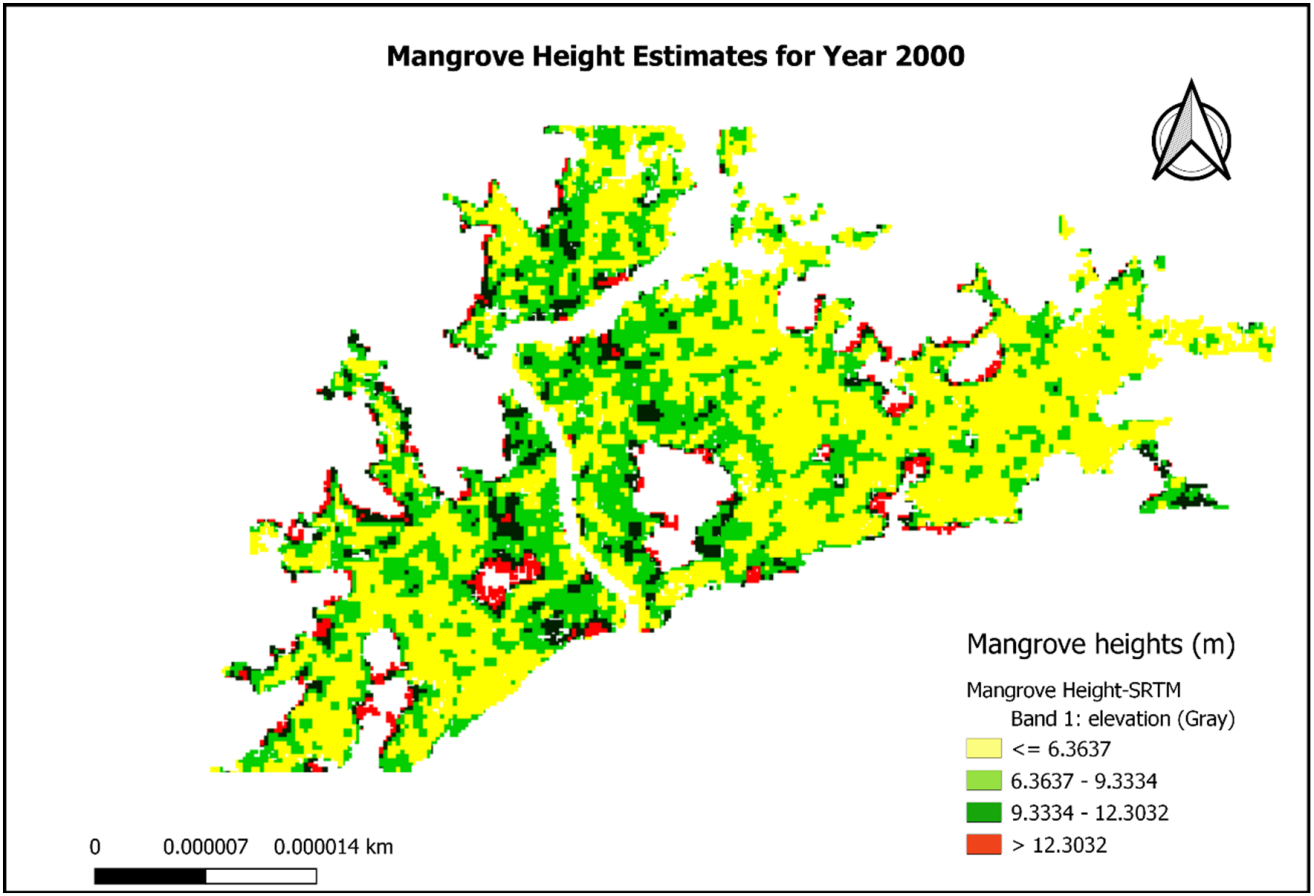
Mangrove height estimates for year 2000.

Tree height, density and basal area are often taken into account when evaluating the structure of a forest; these factors are also required to calculate the amount of aboveground biomass. We created a mangrove canopy height map for the year 2000 using the global mangrove distribution vector file, SRTM DEM, and SAR data. This was then utilized to create estimations of AGB for the same time period. Our analysis of mangrove canopy height distribution shows that in year 2000, mangrove canopy height ranges from about 6.3 to 12.7 m (Figure 9).

**FIGURE 10:**
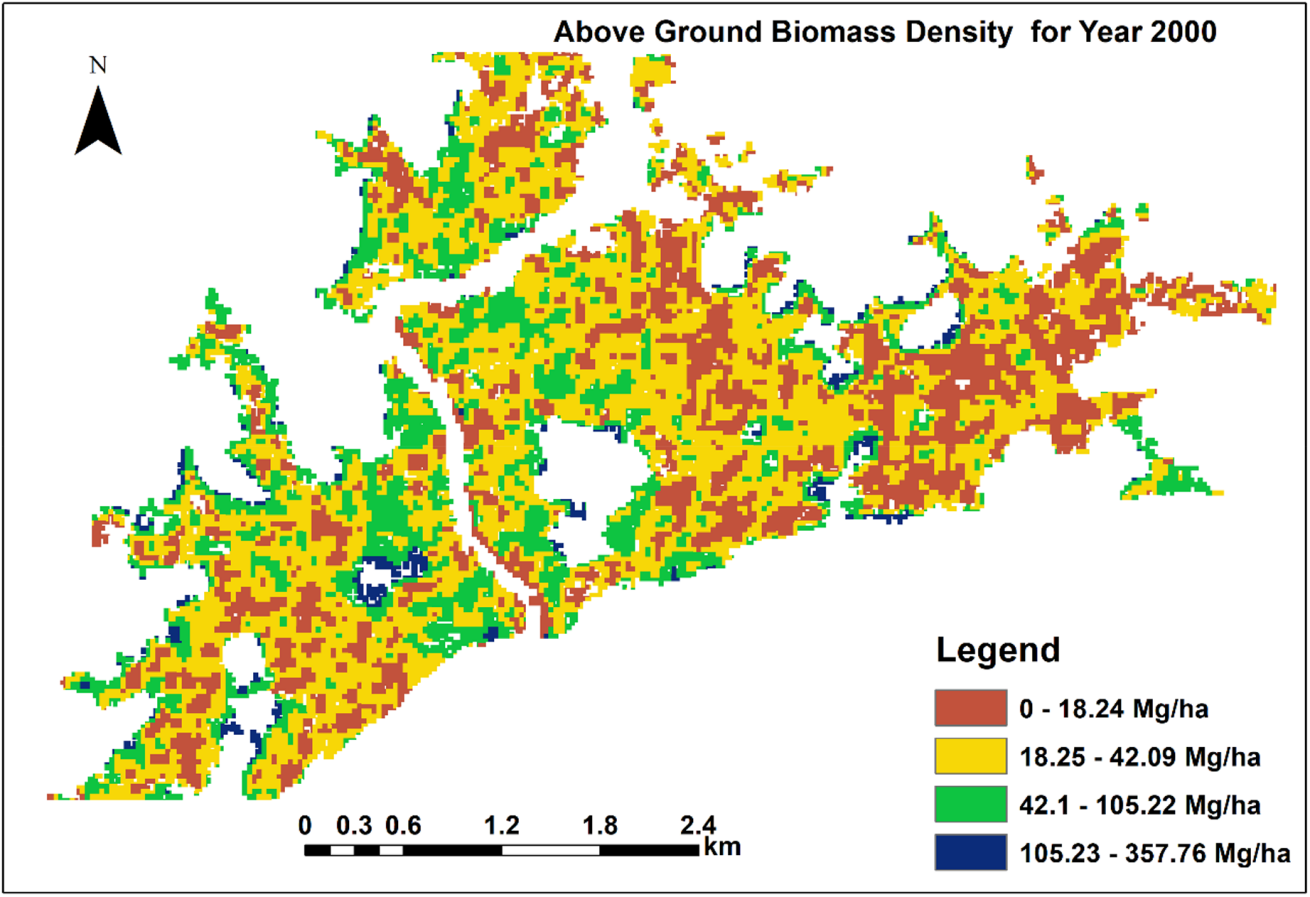
Above ground biomass estimates for year 2000.

This estimate of mangrove height significantly corresponds to previously reported values in different estuaries in Ghana (Aheto et al., 2011 and Nortey et al., 2016). The local‐scale geophysical determinants of environmental gradients, such as nutrient availability and soil or water salinity, are highlighted by the spatial variability in canopy height (Thom, 1982). The spatial distribution of aboveground biomass patterns is determined by the relative influence of local environmental gradients, as well as the spatial distribution of mangroves species in a given coastal region (Rovai et al., 2018). The result of AGB estimate for year 2000 showed that aboveground biomass ranged from 0 to 105 Mg/ha (Figure 10).

### 3.4 Estimates of aboveground biomass from the field

Table 5 below shows the results of AGB calculations using several allometric models. The aboveground biomass observed in the first plot is as follows: 65.33, 39.80 and 87.52 (Mg/ha) for the equation developed by Komiyama et al. (2005); Chave et al. (2005) and Njana et al. (2016), respectively (Table 5). Aboveground biomass ranged from 0 to 87.52 Mg/ha. The AGB estimates generally decreased with distance from the shoreline.

**TABLE 5:** Analysis of AGB (Mg/ha) for the year 2020, generated using three allometric models.

Plots	*H* (m)	DBH (cm)	AGB (Mg/ha) A	AGB (Mg/ha) B	AGB (Mg/ha) C
1	3.2–6.3	1.4–6.48	65.33	39.80	87.52
2	2.3–6.0	0.67–4.91	32.77	19.16	43.40
3	0	0	0	0	0
4	2.5–6.10	4.48	8.75	5.43	11.54

### 3.5 Coastal vulnerability assessment

**FIGURE 11:**
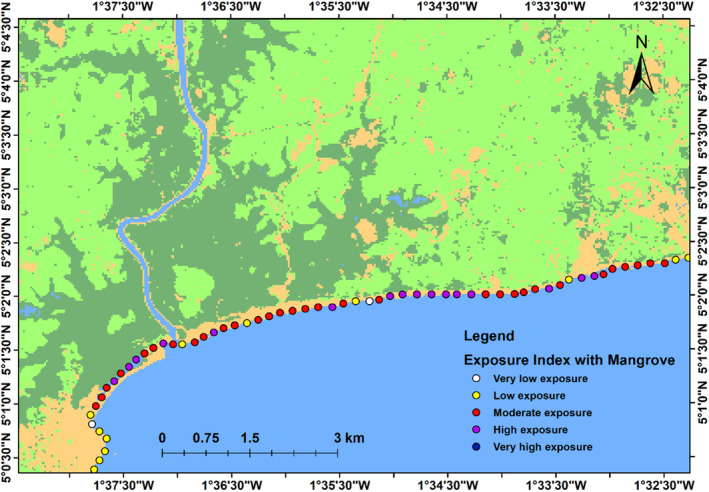
Map of Coastal Exposure Index in the presence of mangrove ecosystem.

**FIGURE 12:**
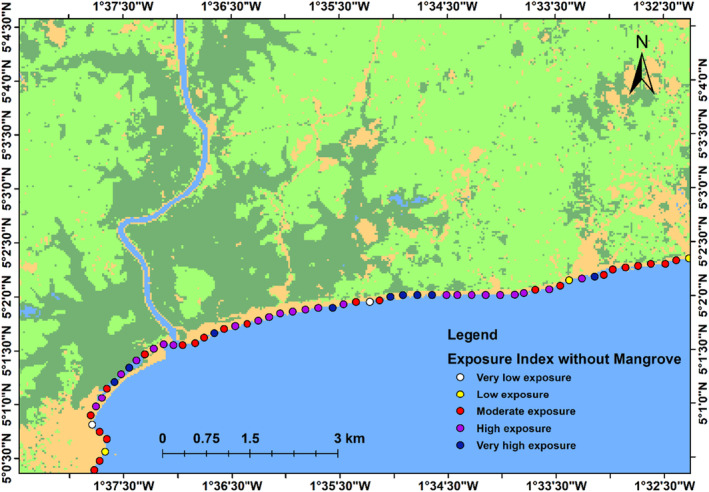
Map of Coastal Exposure Index in the absence of mangrove ecosystem.

We calculate the index of vulnerability for every 250 m along the coast in our study area. This index was used to identify the role of mangrove ecosystem as a nature‐based solution for coastal defence and resilience. The result is presented in Figures 11 and 12, the areas in purple colour denote high‐risk areas, whereas areas in yellow/white colour denote low‐risk areas. Figure 11 showed that in the presence of Mangrove ecosystem, 23% of the coastal segment fall under very low‐/low‐risk zone, 51% of the coastal segment fall under moderate risk, while 26% fall under high‐risk zone. Figure 12 showed that in the absence of Mangrove ecosystem, 8% of the coastal segment fall under low‐risk zone, 38% fall under moderate‐risk zone, 39% fall under high‐risk zone, while 15% fall under very high‐risk zone.

## 4 DISCUSSION

The importance of combining optical and radar data for long‐term coastal ecosystem mapping was highlighted by Mwita et al. (2012), Wijedasa et al. (2012) and Aja et al. (2022). Combining radar and optical imagery allows for effective landcover mapping, and recent developments in artificial intelligence, machine learning algorithms and the advent of platforms for high‐performance cloud computing, such as Google Earth Engine (GEE) have made this possible (Erika et al., 2020; Midekisa et al., 2017; Wijedasa et al., 2012). This gave us the opportunity to thoroughly evaluate the ecosystem of interest. The LULC changes for the study region between 2009 and 2019 are depicted in Figures 4, 5 and 6.

The findings demonstrated that over the course of the two time period, changes occurred in all the LULC. In 2009, mangroves occupied 1613 hectares (ha), followed by 370 ha of bare/built‐up area, 2617 ha of other vegetation, and 1770 ha of water bodies. Mangrove areas decreased in 2019 to 1340 ha, bare/built‐up areas increased to 549 ha, other vegetation areas decreased to 2062 ha, and water body areas increased to 1891 ha equivalent. This suggests that portions of bare/built‐up (which could be agricultural land) have replaced mangroves and other vegetation. A portion of the area that were originally covered by mangroves and other vegetation have also been occupied by water bodies.

To offer thorough statistical analysis for each classification, we made use of confusion matrices and an independent accuracy evaluation. The confusion matrix for the 2009 imagery classification revealed that, of the 647 pixels that were identified as mangroves, all were classified correctly, whereas the confusion matrix for the 2019 image revealed that, of the 647 pixels that were identified as mangroves, 642 pixels were classified correctly as mangroves (Tables 3 and 4). This could be as a result of the effect of using L‐band in 2009 and C‐band in 2019 classification. Similarly, Attarchi and Gloaguen (2014) as well as Aja et al. (2022) noted that L‐band synthetic aperture radar performs better than C‐band when combined with optical data. The strength of the classification method is underscored by the independent accuracy assessment (Figures 7 and 8), which show that the RF algorithm was able to identify the different landcover fairly well. The overall accuracy for 2009 classification was 99.6% while the overall accuracy for 2019 was 99.1%, demonstrating that SAR L‐band outperforms C‐band. The QADI further demonstrated that the generated maps have a good agreement with the ground truth and that our classification has a good confidence level. This finding suggests that the mangrove extent mapping using a mix of radar and optical data was outstanding. For example, according to Table 1, the QADI for the 2019 classification was 0.00529, whereas the QADI for the 2009 classification was 0.00794, both of which fall within the very high confidence threshold.

In the current study, result of mangrove inventory for the year 2020 showed that the DBH (diameter at breast height) for all mangrove species ranged from 0.67 to 6.48 cm while mangrove height ranged from 2.3 to 6.34 m. This indicates that the structural development of the mangrove forest is low and agrees with the findings of Aheto et al. (2011) and Nortey et al. (2016) at different estuaries in Ghana. There is strong correlation between the height and DBH of the sampled trees. The regression model that combines tree height, wood density and DBH yielded the best estimate of the aboveground biomass, which is consistent with previous research findings (Nortey et al., 2016; Fatoyinbo et al., 2018).

We found that the spatial distribution of mangrove height and AGB density can be estimated using GEE. Our results from satellite estimates showed that in year 2000, mangrove height ranged from 6.3 to 12.7 m while AGB ranged from 0 to 105 Mg/ha (Figures 9 and 10). The maximum height decreased from 12.7 to 6.3 m while AGB density decreased from 105 to 87.5 mg/ha between year 2000 and 2020. This shows that mangrove height and aboveground biomass have declined in the past 20 years, judging from the results of field work and remote sensing. The primary motivation for using GEE in this investigation was to explore its capability to estimate AGB using ALOS PALSAR and SRTM data. Previously, estimation of AGB involves highly complex computations requiring python or java codes in MATLAB software (Lagomasino et al., 2016; Qi & Dubayah, 2016).

Our analyses delivered a risk reduction map due to the presence of mangrove ecosystem and show where mangrove ecosystem conservation and restoration have the best potential of protecting coastal communities. We developed a hazard index that integrates the protective function of ecosystems for the coastline at a 250‐m scale to identify where mangroves have the best potential to safeguard coastal populations against erosion and flooding. We assessed coastal vulnerability under two scenarios: (1) with mangrove ecosystem and (2) without mangrove ecosystem. Our result shows that with intact mangrove ecosystem, the very low/low (rank = 1 & 2) risk areas represented 23% of the coastal segment, the moderate (rank = 3) risk areas represented 51%, whereas the high‐risk areas (rank = 4) represented 26% (Figure 11). In the absence of mangrove ecosystem as shown in Figure 12, very low‐/low‐risk areas (rank = 1 & 2) represented 8%, moderate‐risk areas (rank = 3) represented 38%, high‐risk areas (rank = 4) represented 39%, while very high‐risk areas (rank = 5) represented 15%. As shown in Figures 11 and 12, coastal vulnerability will increase if mangrove forest is removed. This result agrees with the findings of Arkema et al. (2013) and Al Ruheili and Boluwade (2023).

## 5 CONCLUSION

The rate of tropical and subtropical mangrove forest degradation is increasing, and this is increasing the chance that coastal disasters will harm more people and property. Consequently, there is a critical need to map and keep track of this ecosystem and to identify their multiple benefits and values in the context of coastal resilience. In this study, we mapped and inventoried a complex mangrove wetland based on satellite data from multiple sources, secondary data sets, state of the art modelling tools and appraised the impacts of anthropogenic activities on their temporal and spatial changes. By so doing, we identified that the mangrove ecosystem is being converted to other land uses, which is also leading to a decline in the AGB density and these patterns of change can help guide conservation efforts and enable sustainable usage of our priceless mangrove ecosystem.

Knowing where mangroves are most likely to lessen vulnerability to erosion and floods from storms and future sea levels, as well as safeguard vulnerable people and property, is necessary to prioritize mangrove habitats for conservation or restoration in service of natural hazard reduction.

We developed an index of hazard that takes into account the protective function of mangrove ecosystems at every 250‐m scale in order to pinpoint the coastline segments where mangroves have the greatest potential to safeguard coastal communities against hazard. We evaluated the vulnerability of the coastline with and without mangrove ecosystem for the entire coastline of 593 points along the coastal segment. We found that in the presence of mangrove, about 74% of the coastal segment fall under low‐/moderate‐risk zone, which decreased to 46% without mangrove.

For prioritizing mangroves for coastal defense, the vulnerability index from this paper is most helpful. Our findings highlighted that continuous degradation of existing mangrove ecosystems will lead to greater harm to people and property. We therefore recommend that coastal defense planning in Ghana must incorporate mangrove ecosystems restoration alongside physical structures.

The accuracy of our result is within the limited bounds of the data spatial resolution. A major limitation of this study is the lack of up‐to‐date images (e.g. 2020 images) and high‐resolution images (e.g. 10‐m resolution) in GEE database for the study region as at the time of this analysis. The coastal vulnerability suit in the InVEST model serves as an effective tool for rapid assessment of habitat scenarios in the context of coastal resilience to inform decision‐makers. However, the model has some limitations because of the use of a surge proxy that could lead to a generalization of storm dynamics and may result in the overestimation or underestimation of risks for some coastal areas. Future study will extend the methodology to the entire coastline of Ghana, incorporating the impacts of sea level rise.

## AUTHOR CONTRIBUTIONS

**Daniel Aja:** Conceptualization (lead); data curation (lead); formal analysis (lead); funding acquisition (lead); investigation (lead); methodology (lead); software (lead); validation (lead); visualization (lead); writing – original draft (lead). **Michael Miyittah:** Conceptualization (supporting); formal analysis (supporting); funding acquisition (equal); methodology (supporting); project administration (lead); supervision (lead); writing – review and editing (lead). **Donatus Bapentire Angnuureng:** Conceptualization (supporting); formal analysis (supporting); funding acquisition (equal); methodology (supporting); software (supporting); supervision (supporting); writing – review and editing (supporting).

## FUNDING INFORMATION

This project was supported by the World Bank in cooperation with the government of Ghana via the Africa Center of Excellence in Coastal Resilience (ACECoR) [Credit No.: 6389‐GH].

## CONFLICT OF INTEREST STATEMENT

We have no conflicting interest to declare.

## Data Availability

The links to the data sets analysed during this study are provided in Section 2.2. The codes used for this study are available at https://doi.org/10.5061/dryad.n2z34tn2d.
